# The significant influence of having children on the postoperative prognosis of patients with nonsmall cell lung cancer: A propensity score‐matched analysis

**DOI:** 10.1002/cam4.1539

**Published:** 2018-05-29

**Authors:** Shinkichi Takamori, Tetsuzo Tagawa, Gouji Toyokawa, Hiroki Ueo, Mototsugu Shimokawa, Fumihiko Kinoshita, Taichi Matsubara, Yuka Kozuma, Naoki Haratake, Takaki Akamine, Masakazu Katsura, Kazuki Takada, Fumihiko Hirai, Fumihiro Shoji, Tatsuro Okamoto, Yoshinao Oda, Yoshihiko Maehara

**Affiliations:** ^1^ Department of Surgery and Science Graduate School of Medical Sciences Kyushu University Fukuoka Japan; ^2^ Department of Surgery Saiseikai Fukuoka General Hospital Fukuoka Japan; ^3^ Clinical Research Institute National Kyushu Cancer Center Minami‐ku Fukuoka Japan; ^4^ Department of Anatomic Pathology Graduate School of Medical Sciences Kyushu University Fukuoka Japan

**Keywords:** cancer survivor, nonsmall cell lung cancer, nutrition, prognosis, surgery

## Abstract

The aim of this study was to elucidate the relationship between family‐associated factors and the postoperative prognosis in patients with nonsmall cell lung cancer (NSCLC). Additionally, we investigated whether having children was associated with the postoperative maintenance of the nutritional status. We selected 438 NSCLC patients who had undergone curative lung resection between 2004 and 2011 at Kyushu University (Fukuoka, Japan), whose family‐associated factors were available. Nutritional indices, including the prognostic nutritional index (PNI), were used to estimate the change in the nutritional status for 1 year after surgery. A propensity score analysis was conducted after adjusting the following variables: sex, age, smoking history, performance status, pathological stage, and histological type. Three hundred patients (68.5%) had both children and partners. Forty‐nine patients (11.2%) only had children, and 56 (12.8%) patients only had a partner. Thirty‐three patients (7.5%) did not have a partner or children. The overall survival (OS) and disease‐free survival (DFS) of the partner‐present and partner‐absent patients did not differ to a statistically significant extent (*P *=* *.862 and *P *=* *.712, respectively). However, childless patients showed significantly shorter OS and DFS in comparison with patients with children (*P *=* *.005 and *P *=* *.002, respectively). The postoperative exacerbation of PNI was significantly greater in childless patients than in patients with children (*P *=* *.003). These results remained after propensity score matching. Childless patients had a significantly poorer postoperative prognosis than those with children. Surgeons caring for childless NSCLC patients should be aware of the poorer postoperative outcomes in this population.

## 1 INTRODUCTION

Lung cancer remains one of the main causes of cancer death worldwide.^1^ For several decades, epidemiologists have been exhaustively investigating the epidemiology of lung cancer.^2^ Efforts to elucidate the association between cigarette smoking and lung cancer have been chronicled,^3^ and a previous study supported the association between physical activity and the risk of lung cancer.^4^ In addition, Menvielle et al^5^ focused on the socioeconomic gradient of lung cancer occurrence. Epidemiological studies have also identified that occupational exposure to agents such as asbestos, radon, and silica is associated with a risk of lung cancer or mortality.^6^, ^7^, ^8^ These previous studies have highlighted the need for public health policies aimed at reducing the number of lung cancer patients.

A number of recent epidemiological studies have shown that family‐associated factors, including having partner and/or children, were significantly associated with survival.^9^, ^10^, ^11^, ^12^ Regarding patients with cancer, previous studies have investigated the relationship between family‐associated factors and the incidence of (or deaths from) various types of malignancy.^13^, ^14^, ^15^, ^16^, ^17^ However, whether or not having children influences the pathogenesis or prognosis of solid organ malignancies remains controversial. For example, a previous report found that the mortality rate of married male cancer patients with children was one‐third lower than that of childless and never‐married patient.^13^ Another study suggested that having children beneficially influenced the incidence of Hodgkin’s disease.^14^ In contrast, the number of children was found to have no influence on the survival of patients with colorectal cancer.^15^, ^16^ In lung cancer patients specifically, a previous meta‐analysis did not show a significant relationship between the presence or absence of children and the risk of lung cancer.^17^ However, the influence of having children on the postoperative prognosis remains unclear. Although our previous study has suggested that childless patients may be related to poor postoperative prognosis,^18^ the definitive conclusion could not be drawn due to the bias of retrospective nature. Therefore, this study explored the relationship between family‐associated factors and the postoperative prognosis of patients with nonsmall cell lung cancer (NSCLC) using larger sample size and propensity score‐matched analysis. In addition, according to a previous article, social support from children plays a pivotal role in maintaining the physical and mental health of the elderly people.^19^ Thus, in this study, we hypothesized that having children would influence the postoperative nutritional status and investigated the association between the family‐associated factors and postoperative change in the nutritional status.

## 2 MATERIALS AND METHODS

### 2.1 Patients and methods

From January 2004 to December 2011, 480 patients underwent the complete resection of primary NSCLC at the Department of Surgery and Science, Graduate School of Medical Sciences, Kyushu University (Fukuoka, Japan). Among these, we excluded 42 patients whose family‐associated factors were not available. Thus, the data of the remaining 438 patients were included in the analysis. The gender, age, smoking history (pack year index: PY), histopathology, performance status (PS), adjuvant chemotherapy, and surgical procedure were investigated. The 7th edition of the TNM Classification of Malignant Tumors was used for determining the pathological stage. The blood analysis results, including the lymphocyte count, and the albumin, cholesterol, and C‐reactive protein levels were also analyzed within 1 month before surgery and at approximately 1 year (10‐14 months) after surgery. The institutional review board (IRB) of our institution approved this study (IRB No. 29‐260).

### 2.2 Analyzing the postoperative change in the nutritional status

In order to investigate the nutritional status, the prognostic nutritional index (PNI), controlling nutritional status (CONUT), and modified Glasgow prognostic score (mGPS) were calculated as previously described.^20^, ^21^, ^22^ In brief, the PNI was calculated as follows: 10 × albumin + 0.005 × lymphocyte count.^20^ The CONUT score was defined as shown in Table S1.^21^ The mGPS value was defined as shown in Table S2.^22^ The change in each nutritional index during the year after surgery was expressed as ΔPNI/y, ΔCONUT/y, and ΔmGPS/y. The decrease in the PNI and the increase in the CONUT and mGPS values indicate the exacerbation of the nutritional status.

### 2.3 Statistical analyses

The associations between the family‐associated factors and clinical factors were analyzed using Student’s *t* test or the Mann‐Whitney *U* test for continuous variables. For categorical variables, the relationships between the family‐associated factors and clinical factors were investigated using Pearson’s chi‐square test or Fisher’s exact 2‐sided test. Overall survival (OS) was defined as the time (months) from the day of the operation until death from any cause. Disease‐free survival (DFS) was defined as the time (months) from the day of the operation until recurrence or death from any cause. The Kaplan‐Meier method and Wilcoxon’s test were used to estimate the probability of survival. Cox’s proportional hazards models were used to calculate the hazard ratio. *P* values of <.05 were considered to indicate statistical significance. All of the analyses were performed using the software program JMP^®^, version 13 (SAS Institute Inc., Cary, NC).

### 2.4 Propensity score matching

A propensity score analysis was conducted with the aim of reducing the bias of the retrospective nature of the study. The propensity scores, which were calculated by a multivariable logistic analysis, included the following variables: sex, age, smoking history, PS, pStage, and histological type. A propensity score difference of 0.05 was adopted as the maximum caliper width for matching both the child‐present and childless groups. Finally, 89 matched patients from each group were included in the analysis. Similarly, 74 matched patients from partner‐present and partner‐absent groups were analyzed.

## 3 RESULTS

### 3.1 Characteristics of the enrolled patients

The patient characteristics are shown in Table S3. The median age was 69 years (range 29‐93 years), and 247 patients (56.4%) were men. Two hundred and fifty‐three patients (57.8%) were current or former smokers (median PY, 20; range 0‐165). Three hundred and ten patients (70.7%), 67 patients (15.3%), and 61 patients (14.0%) were diagnosed with pathological stages I, II, and III, respectively; 328 patients (74.9%) were diagnosed with adenocarcinoma. Almost all of the patients (97.0%) had a PS of 0 or 1. One hundred and fifteen patients (26.3%) received adjuvant chemotherapy. Three hundred and fifty‐six patients (81.3%) underwent lobectomy or pneumonectomy. Three hundred and forty‐nine patients (79.7%) had children; 89 (25.5%) patients had 1 child, 166 (47.6%) had 2 children, 75 (21.5%) had 3 children, and 19 (5.4%) had 4 children. Three hundred and fifty‐six patients (81.3%) had partners. Three hundred patients (68.5%) had both children and partners. Forty‐nine patients (11.2%) only had children, and 56 (12.8%) only had a partner. Thirty‐three patients (7.5%) had neither children nor a partner. Table 1 shows the relationship between the children and partner statuses. The presence or absence of children was significantly associated with the presence or absence of a partner (*P *<* *.001).

**Table 1:** The relationship between children and partner statuses in patients with NSCLC

Factors	Partner		*P* value
	Present (n = 356)	Absent (n = 82)	
Children			
Present (n = 349)	300 (86.0%)	49 (14.0%)	<.001
Absent (n = 89)	56 (62.9%)	33 (37.1%)	

NSCLC, nonsmall cell lung cancer.

### 3.2 Baseline characteristic of child‐present and child‐absent patients with NSCLC

Table 2 shows the baseline characteristic of child‐present and childless NSCLC patients who had undergone curative lung resection. There were no significant differences between the child‐present and childless groups with regard to age (*P *=* *.343), sex (*P *=* *.403), smoking status (*P *=* *.550), PS (*P *=* *.533), pStage (*P *=* *.337), histological type (*P *=* *.710), and adjuvant chemotherapy (*P *=* *.636). Propensity score matching was performed as described in the statistical methods. The 89 matched patients from the child‐present group and 89 matched patients from the childless group were included in the propensity score‐matched analysis. After propensity score matching, the distribution of the baseline patient characteristics between the child‐present and childless groups was well balanced (Table 2). Table 3 shows the baseline characteristic of partner‐present and partner‐absent patients. The partner‐absent patients were significantly associated with older age (*P *=* *.027) and female sex (*P *<* *.001). There were no significant differences between the partner‐present and partner‐absent patients with regard to smoking status (*P *=* *.137), PS (*P *=* *.586), pStage (*P *=* *.574), histological type (*P *=* *.952), and adjuvant chemotherapy (*P *=* *.314). After propensity score matching, the distribution of the baseline patient characteristics between the partner‐present and partner‐absent groups was well balanced (Table 3).

**Table 2:** Baseline characteristics of child‐present and child‐absent patients with NSCLC

Factors		Before propensity score matching			After propensity score matching		
		Child present (n = 349)	Child absent (n = 89)	*P* value	Child present (n = 89)	Child absent (n = 89)	*P* value
Age (years)	Mean (SD)	67.7 (9.9)	66.5 (9.9)	.343	66.0 (10.8)	66.5 (9.9)	.717
Sex, n (%)	Men	193 (55.3%)	54 (60.7%)	.403	55 (61.8%)	54 (60.7%)	1.000
	Women	156 (44.7%)	35 (39.3%)		34 (38.2%)	35 (39.3%)	
Smoking, n (%)	Never	150 (43.0%)	35 (39.3%)	.550	34 (38.2%)	35 (39.3%)	1.000
	Ever	199 (57.0%)	54 (60.7%)		55 (61.8%)	54 (60.7%)	
Performance status, n (%)	0	260 (74.5%)	66 (74.2%)	.533	70 (78.7%)	66 (74.2%)	.656
	1	80 (22.9%)	19 (21.3%)		17 (19.1%)	19 (21.4%)	
	2	7 (2.0%)	4 (4.5%)		2 (2.2%)	4 (4.4%)	
	3	2 (0.6%)	0 (0%)		0 (0%)	0 (0%)	
Pathological stage, n (%)	IA	185 (53.0%)	36 (40.5%)	.337	35 (39.3%)	36 (40.5%)	.919
	IB	68 (19.5%)	21 (23.6%)		24 (27.0%)	21 (23.6%)	
	IIA	18 (5.2%)	6 (6.7%)		7 (7.9%)	6 (6.7%)	
	IIB	32 (9.2%)	11 (12.4%)		12 (13.5%)	11 (12.4%)	
	IIIA	44 (12.6%)	15 (16.8%)		11 (12.3%)	15 (16.8%)	
	IIIB	2 (0.5%)	0 (0%)		0 (0%)	0 (0%)	
Histological type, n (%)	Ad	264 (75.6%)	64 (71.9%)	.710	64 (71.9%)	64 (71.9%)	1.000
	Sq	66 (18.9%)	19 (21.4%)		20 (22.5%)	19 (21.4%)	
	Others	19 (5.5%)	6 (6.7%)		5 (5.6%)	6 (6.7%)	
Adjuvant chemotherapy, n (%)^a^	Present	82 (50.0%)	29 (54.7%)	.636	26 (48.1%)	29 (54.7%)	.564
	Absent	82 (50.0%)	24 (45.3%)		28 (51.9%)	24 (45.3%)	

NSCLC, nonsmall cell lung cancer; SD, standard deviation; Ad, adenocarcinoma; Sq, squamous cell carcinoma.

Cases in which adjuvant chemotherapy was indicated.

**Table 3:** Baseline characteristics of partner‐present and partner‐absent patients with NSCLC

Factors		Before propensity score matching			After propensity score matching		
		Partner present (n = 356)	Partner absent (n = 82)	*P* value	Partner present (n = 74)	Partner absent (n = 74)	*P* value
Age (years)	Mean (SD)	66.9 (9.9)	69.6 (9.9)	.027	67.5 (10.9)	68.8 (9.7)	.466
Sex, n (%)	Men	216 (60.7%)	31 (37.8%)	<.001	28 (37.8%)	31 (41.9%)	.737
	Women	140 (39.3%)	51 (62.2%)		46 (62.2%)	43 (58.1%)	
Smoking, n (%)	Never	144 (40.5%)	41 (50.0%)	.137	33 (44.6%)	36 (48.7%)	.742
	Ever	212 (59.5%)	41 (50.0%)		41 (55.4%)	38 (51.3%)	
Performance status, n (%)	0	264 (74.2%)	62 (75.6%)	.586	59 (79.7%)	57 (77.0%)	.692
	1	83 (23.3%)	16 (19.5%)		13 (17.6%)	14 (18.9%)	
	2	8 (2.2%)	3 (3.7%)		2 (2.7%)	2 (2.7%)	
	3	1 (0.3%)	1 (1.2%)		0 (0%)	1 (1.4%)	
Pathological stage, n (%)	IA	182 (51.1%)	39 (47.5%)	.574	42 (56.8%)	38 (51.4%)	.933
	IB	71 (20.0%)	18 (22.0%)		12 (16.2%)	15 (20.3%)	
	IIA	20 (5.6%)	4 (4.9%)		4 (5.4%)	4 (5.4%)	
	IIB	31 (8.7%)	12 (14.6%)		9 (12.2%)	8 (10.8%)	
	IIIA	50 (14.0%)	9 (11.0%)		7 (9.4%)	9 (12.1%)	
	IIIB	2 (0.6%)	0 (0%)		0 (0%)	0 (0%)	
Histological type, n (%)	Ad	266 (74.7%)	62 (75.6%)	.952	57 (77.0%)	58 (78.4%)	.976
	Sq	70 (19.7%)	15 (18.3%)		13 (17.6%)	12 (16.2%)	
	Others	20 (5.6%)	5 (6.1%)		4 (5.4%)	4 (5.4%)	
Adjuvant chemotherapy, n (%)^a^	Present	92 (52.9%)	19 (44.2%)	.314	11 (34.4%)	17 (47.2%)	.330
	Absent	82 (47.1%)	24 (55.8%)		21 (65.6%)	19 (52.8%)	

NSCLC, nonsmall cell lung cancer; SD, standard deviation; Ad, adenocarcinoma; Sq, squamous cell carcinoma.

a Cases in which adjuvant chemotherapy was indicated.

### 3.3 Influence of family‐associated factors on the postoperative prognosis

**Figure 1:**
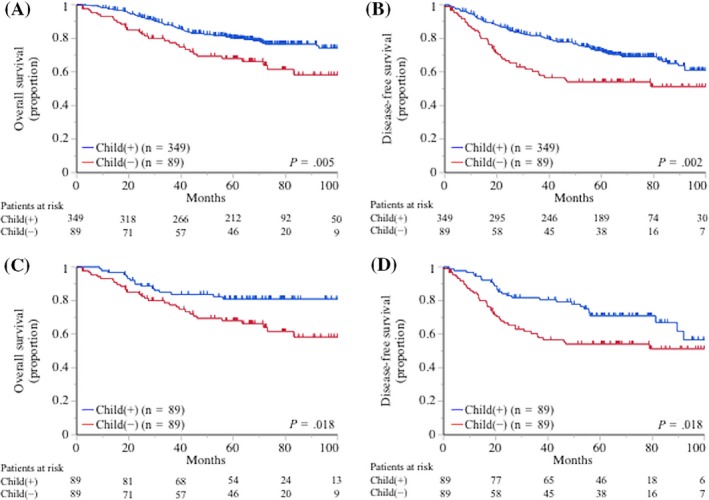
The Kaplan‐Meier curves for the (A) overall survival (OS) and (B) disease‐free survival (DSF) for 438 patients according to the presence or absence of children are shown (*P *=* *.005 and *P *=* *.002, respectively). After propensity matching, the childless group had a significantly shorter (C) OS and (D) DFS than the child‐present group (*P *=* *.018 and *P *=* *.018, respectively)

**Figure 2:**
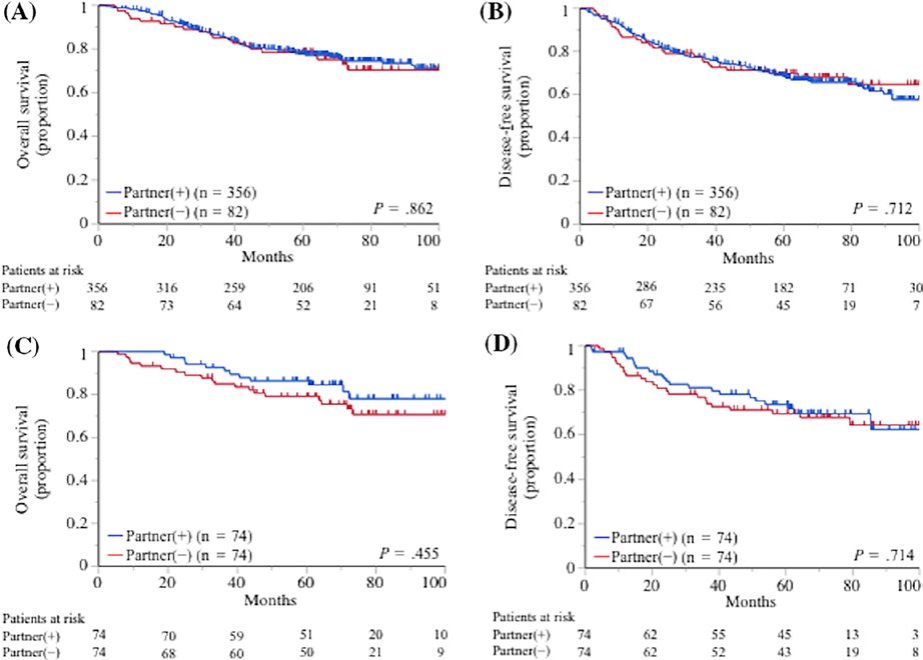
The Kaplan‐Meier curves for the (A) overall survival (OS) and (B) disease‐free survival (DSF) for 438 patients according to the presence or absence of a partner are shown. The (A) OS and (B) DFS of the groups did not differ to a statistically significant extent (*P *=* *.862 and *P *=* *.712, respectively). After propensity matching, the (C) OS and (D) DFS of the groups did not differ to a statistically significant extent (*P *=* *.455 and *P *=* *.714, respectively)

Childless patients had a significantly shorter OS in comparison with patients with children (77.4% vs 87.9% at 3 years; hazard ratio for death, 1.79; 95% confidence interval, 1.22‐2.56; *P *=* *.003; Figure 1A). Childless patients also had a significantly shorter DFS in comparison with patients with children (60.4% vs 81.6% at 3 years; hazard ratio for disease progression or death, 1.81; 95% confidence interval, 1.17‐2.74; *P *=* *.009; Figure 1B). After propensity score matching, childless patients had a significantly shorter OS in comparison with patients with children (77.4% vs 84.5% at 3 years; hazard ratio for death, 2.03; 95% confidence interval, 1.13‐3.75; *P *=* *.017; Figure 1C). Childless patients also had a significantly shorter DFS in comparison with patients with children (60.4% vs 81.7% at 3 years; hazard ratio for disease progression or death, 1.79; 95% confidence interval, 1.10‐2.96; *P *=* *.018; Figure 1D). The number of children did not affect the postoperative OS or DFS (*P *=* *.696 and *P *=* *.578, respectively; Figure S1). The OS and DFS of the partner‐present and partner‐absent patients did not differ to a statistically significant extent (*P *=* *.862 and *P *=* *.712, respectively; Figure 2A,B). This finding remained after propensity score matching (Figure 2C,D).

### 3.4 Impact of family‐associated factors on the postoperative change in the nutritional status

Tables 4 and 5 show the influence of family‐associated factors on the postoperative change in the PNI, CONUT, and mGPS values during the year after surgery. Of note, a decrease in the PNI and an increase in the CONUT or mGPS values indicated the exacerbation of the nutritional status. As shown in Table 4, the postoperative exacerbation of PNI, CONUT, and mGPS values was significantly greater in the childless group than in the child‐present group (*P *=* *.003, *P *=* *.001, and *P *<* *.001, respectively). After propensity score matching, the postoperative exacerbation of the PNI, CONUT, and mGPS values was also significantly greater in the childless group (*P *=* *.002, *P *=* *.032, and *P *=* *.001, respectively). As shown in Table 5, the postoperative change in the PNI, CONUT, and mGPS values of the partner‐present and partner‐absent patients did not differ to a statistically significant extent (*P *=* *.567, *P *=* *.428, and *P *=* *.701, respectively). This result remained significant after propensity score matching.

**Table 4:** The association between the postoperative change in the nutritional indices and the presence or absence of children in patients with NSCLC

Nutritional indices^a^		Before propensity score matching			After propensity score matching		
		Child present (n = 317)	Child absent (n = 69)	*P* value	Child present (n = 77)	Child absent (n = 69)	*P* value
ΔPNI/y	Mean (SD)	−8.44 (4.87)	−10.44 (5.28)	.003	−7.93 (4.38)	−10.44 (5.28)	.002
ΔCONUT/y	Mean (SD)	0.00 (1.35)	0.58 (1.32)	.001	0.10 (1.32)	0.58 (1.32)	.032
ΔmGPS/y	Mean (SD)	−0.05 (0.41)	0.16 (0.56)	<.001	−0.10 (0.42)	0.16 (0.56)	.001

NSCLC, nonsmall cell lung cancer; SD, standard deviation; Δ, the difference between preoperative and postoperative (1 year after surgery) indices; PNI, prognostic nutritional index; CONUT, controlling nutritional status; mGPS, modified Glasgow prognostic score.

a The decrease in the PNI and the increase in the CONUT or mGPS values indicate the exacerbation of the nutritional status.

**Table 5:** The association between the postoperative change in the nutritional indices and the presence or absence of partner in patients with NSCLC

Nutritional indices^a^		Before propensity score matching			After propensity score matching		
		Partner present (n = 312)	Partner absent (n = 74)	*P* value	Partner present (n = 74)	Partner absent (n = 74)	*P* value
ΔPNI/y	Mean (SD)	−9.57 (9.38)	−10.21 (11.04)	.594	−8.73 (7.13)	−9.44 (10.27)	.480
ΔCONUT/y	Mean (SD)	0.49 (2.39)	0.52 (2.65)	.913	0.34 (2.04)	0.41 (2.49)	.857
ΔmGPS/y	Mean (SD)	−0.01 (0.45)	−0.02 (0.22)	.757	−0.05 (0.37)	−0.03 (0.23)	.593

NSCLC, nonsmall cell lung cancer; SD, standard deviation; Δ, the difference between preoperative and postoperative (1 year after surgery) indices; PNI, prognostic nutritional index; CONUT, controlling nutritional status; mGPS, modified Glasgow prognostic score.

a The decrease in the PNI and the increase in the CONUT or mGPS values indicate the exacerbation of the nutritional status.

## 4 DISCUSSION

In the current study, we showed that the childless NSCLC patients had a significantly poorer postoperative prognosis in comparison with the patients with children (Figure 1), although the number of children did not influence the postoperative prognosis (Figure S1). According to a previous study, the emotional support provided by children plays a pivotal role in maintaining the physical and mental health of the elderly people.^19^ Given that the median age at the diagnosis of lung cancer is 70 years,^23^ patients with NSCLC may benefit from emotional support from their children. The majority of NSCLC patients have a decreased physical function after lung resection^24^; thus, childless patients may need careful social support after surgery. In addition, we hypothesized that emotional support from children contributed to the patients’ nutritional condition and investigated the postoperative change in the patients’ nutritional status using well‐known indices.^20^, ^21^, ^22^ As shown in Table 4, the postoperative exacerbation of the nutritional indices, including the PNI, CONUT, and mGPS, in the childless group was significantly greater than that in the child‐present group. These nutritional indices and the change in the nutritional status have been reported to be significant prognostic factors in various types of malignancies (including lung cancer),^20^, ^21^, ^22^, ^25^ and this evidence would partly explain the reason why having children significantly influenced the postoperative prognosis of the patients in the present study.

With regard to the association between having a partner and the postoperative prognosis, the prognosis and nutritional status of the partner‐present and partner‐absent patients did not differ to a statistically significant extent (Figure 2 and Table 5). As expected, the absence of partner was significantly related to not having children (Table 1); however, being childless had more clinical impact on the postoperative prognosis than not having a partner. Given that lung cancer is a disease of older age,^23^ the patients’ children may play a more important role as a caregiver than the patient’s partner. However, several reports suggested that the marital status was significantly associated with survival in patients with lung cancer,^26^, ^27^ and our data also showed a slight tendency toward a better prognosis in the partner‐present patients in comparison with the partner‐absent patients after propensity score matching (Figure 2). Thus, further investigation with a larger cohort might reveal the latent relationship between having a partner and a better postoperative prognosis. In addition, despite the relatively substantial evidence to support the association between the marital status and the treatment outcomes in lung cancer patients,^26^, ^27^ there is little evidence of the influence of having children on the post‐treatment prognosis. Further studies should be performed to determine which of these family‐associated factors have the greatest influence.

The present study was associated with several limitations. First, this was a retrospective study with a relatively small population that was performed in a single center in Japan. These points made it difficult to reach definitive conclusions. Although a propensity score‐matched analysis was performed to minimize the bias due to the retrospective nature of the study, the results of this study should be validated in a larger prospective observational study in a well‐defined patient population. Second, the current study did not analyze all the potential confounding factors, including the distance between hospital and home, socioeconomic status, and health conditions of the children and/or partners, because these data were not available in this retrospective study; thus, it is important to validate the findings obtained in this study in future prospective studies investigating other populations.

In conclusion, childless patients with NSCLC had a significantly poorer postoperative prognosis in comparison with patients with children. A childless status was significantly associated with the postoperative exacerbation of the nutritional status. Surgeons caring for NSCLC patients who do not have children should be aware of the poorer postoperative outcomes that were observed in this population. Further prospective studies are required to validate these findings.

## CONFLICT OF INTEREST

The authors have declared no conflict of interest.
