# Supplementary figures and images for: The significant influence of having children on the postoperative prognosis of patients with nonsmall cell lung cancer: A propensity score‐matched analysis
Source: Cancer Med. 2018 May 29;7(7):2860–7. doi: 10.1002/cam4.1539 (PMC6051155; doi:10.1002/cam4.1539)

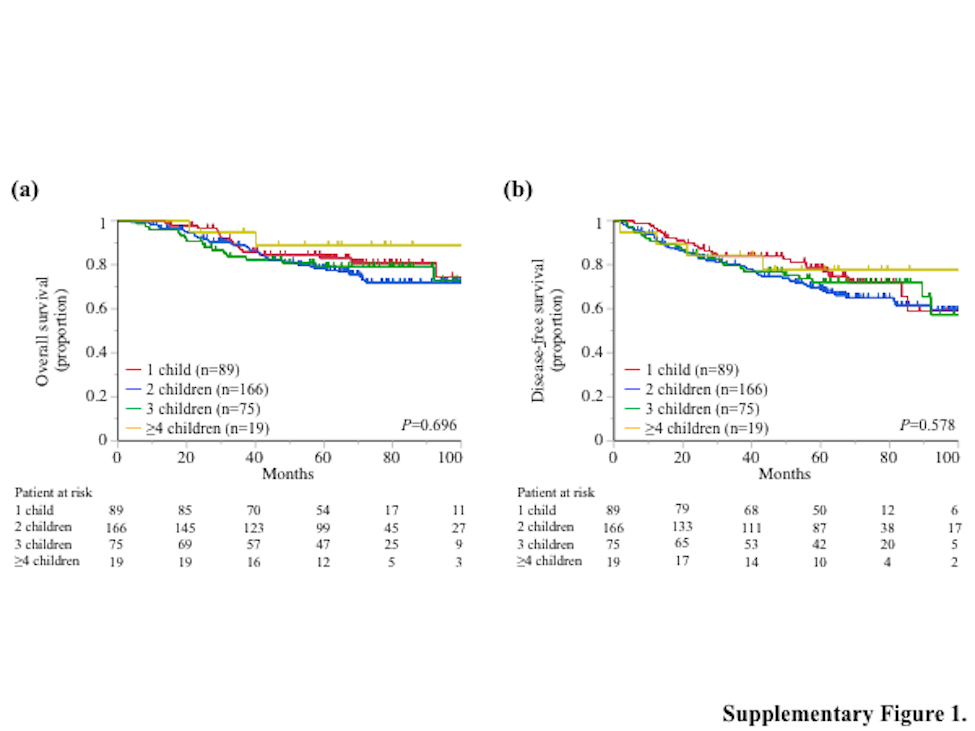

Supplement: Supplementary file 1 [file CAM4-7-2860-s001.tiff]
