# Supplementary material for: The significant influence of having children on the postoperative prognosis of patients with nonsmall cell lung cancer: A propensity score‐matched analysis
Source: Cancer Med. 2018 May 29;7(7):2860–7. doi: 10.1002/cam4.1539 (PMC6051155; doi:10.1002/cam4.1539)
Supplement: Supplementary file 2 [file CAM4-7-2860-s002.docx]

**Supplementary Table 1.** The assessment of the nutritional status by the CONUT score

| **Factors** | **Range and Score** | | | |
| --- | --- | --- | --- | --- |
| Albumin (g/dL) | ≥3.50 | 3.00-3.49 | 2.50-2.99 | <2.50 |
| Score | 0 | 2 | 4 | 6 |
|  | | | | |
| Cholesterol (mg/dL) | ≥180 | 140-179 | 100-139 | <100 |
| Score | 0 | 1 | 2 | 3 |
|  | | | | |
| Lymphocyte count (/mm^3^) | ≥1600 | 1200-1599 | 800-1199 | <800 |
| Score | 0 | 1 | 2 | 3 |
|  | | | | |
| CONUT score = albumin score + cholesterol score + lymphocyte score | | | | |

CONUT controlling nutritional status.
