# Supplementary material for: The significant influence of having children on the postoperative prognosis of patients with nonsmall cell lung cancer: A propensity score‐matched analysis
Source: Cancer Med. 2018 May 29;7(7):2860–7. doi: 10.1002/cam4.1539 (PMC6051155; doi:10.1002/cam4.1539)
Supplement: Supplementary file 3 [file CAM4-7-2860-s003.docx]

**Supplementary Table 2.** The assessment of the nutritional status by the mGPS score

| **Factors** | **mGPS score** |
| --- | --- |
| C-reactive protein≤1.0 (mg/dL) | 0 |
|  | |
| C-reactive protein>1.0 (mg/dL) and albumin≥3.5 (g/dL) | 1 |
|  | |
| C-reactive protein>1.0 (mg/dL) and albumin<3.5 (g/dL) | 2 |

mGPS modified Glasgow prognostic score.
