# Supplementary material for: The significant influence of having children on the postoperative prognosis of patients with nonsmall cell lung cancer: A propensity score‐matched analysis
Source: Cancer Med. 2018 May 29;7(7):2860–7. doi: 10.1002/cam4.1539 (PMC6051155; doi:10.1002/cam4.1539)
Supplement: Supplementary file 4 [file CAM4-7-2860-s004.docx]

**Supplementary Table 3.** The patient characteristics (n = 438)

| **Factors** |  | **Value or no. of patients** |
| --- | --- | --- |
| Age (years) | median (range) | 69 (29–93) |
|  |  |  |
| Sex, n (%) | men | 247 (56.4%) |
|  | women | 191 (43.6%) |
|  |  |  |
| Smoking status, n (%) | never | 185 (42.2%) |
|  | ever | 253 (57.8%) |
|  |  |  |
| Pack years | median (range) | 20 (0-165) |
|  |  |  |
| Pathological stage, n (%) | IA | 220 (50.2%) |
|  | IB | 90 (20.5%) |
|  | IIA | 21 (4.8%) |
|  | IIB | 46 (10.5%) |
|  | IIIA | 59 (13.5%) |
|  | IIIB | 2 (0.5%) |
|  |  |  |
| Histological type, n (%) | adenocarcinoma | 328 (74.9%) |
|  | squamous cell carcinoma | 85 (19.4%) |
|  | others | 25 (5.7%) |
|  |  |  |
| Performance status, n (%) | 0 | 326 (74.4%) |
|  | 1 | 99 (22.6%) |
|  | 2 | 11 (2.5%) |
|  | 3 | 2 (0.5%) |
|  |  |  |
| Surgical procedure, n (%) | ≥lobectomy | 356 (81.3%) |
|  | segmentectomy | 35 (8.0%) |
|  | partial resection | 47 (10.7%) |
|  |  |  |
| Adjuvant chemotherapy, n (%) | none | 323 (73.7%) |
|  | UFT | 47 (10.7%) |
|  | S-1 | 23 (5.3%) |
|  | cisplatin-based | 23 (5.3%) |
|  | carboplatin-based | 16 (3.7%) |
|  | others | 6 (1.3%) |
|  |  |  |
| Children, n (%) | present | 349 (79.7%) |
|  | absent | 89 (20.3%) |
|  |  |  |
| Number of children, n (%) | 1 | 89 (25.5%) |
|  | 2 | 166 (47.6%) |
|  | 3 | 75 (21.5%) |
|  | 4 | 19 (5.4%) |
|  |  |  |
| Partner, n (%) | present | 356 (81.3%) |
|  | absent | 82 (18.7%) |
|  |  |  |
| Children/Partner, n (%) | both | 300 (68.5%) |
|  | only children | 49 (11.2%) |
|  | only partner | 56 (12.8%) |
|  | none | 33 (7.5%) |

SD standard deviation, BMI body mass index, UFT tegafur-uracil, S-1 tegafur-gimeracil-oteracil.
